# Flagellin From *Pseudomonas aeruginosa* Modulates SARS-CoV-2 Infectivity in Cystic Fibrosis Airway Epithelial Cells by Increasing TMPRSS2 Expression

**DOI:** 10.3389/fimmu.2021.714027

**Published:** 2021-12-07

**Authors:** Manon Ruffin, Jeanne Bigot, Claire Calmel, Julia Mercier, Maëlle Givelet, Justine Oliva, Andrés Pizzorno, Manuel Rosa-Calatrava, Harriet Corvol, Viviane Balloy, Olivier Terrier, Loïc Guillot

**Affiliations:** ^1^ Sorbonne Université, Inserm, Centre de Recherche Saint-Antoine (CRSA), Paris, France; ^2^ Laboratoire de Parasitologie-Mycologie, APHP, Hôpital Saint-Antoine, Paris, France; ^3^ CIRI, Centre International de Recherche en Infectiologie, Team VirPath, Université de Lyon, Inserm U1111, Université Claude Bernard Lyon 1, CNRS, UMR5308, ENS de Lyon, Lyon, France; ^4^ Pneumologie Pédiatrique, APHP, Hôpital Trousseau, Paris, France

**Keywords:** cystic fibrosis, infection, COVID19, *Pseudomonas aeruginosa*, SARS-CoV-2, TLR5, protease

## Abstract

In the coronavirus disease 2019 (COVID-19) health crisis, one major challenge is to identify the susceptibility factors of severe acute respiratory syndrome-coronavirus-2 (SARS-CoV-2) in order to adapt the recommendations for populations, as well as to reduce the risk of COVID-19 development in the most vulnerable people, especially patients with chronic respiratory diseases such as cystic fibrosis (CF). Airway epithelial cells (AECs) play a critical role in the modulation of both immune responses and COVID-19 severity. SARS-CoV-2 infects the airway through the receptor angiotensin-converting enzyme 2, and a host protease, transmembrane serine protease 2 (TMPRSS2), plays a major role in SARS-CoV-2 infectivity. Here, we show that *Pseudomonas aeruginosa* increases TMPRSS2 expression, notably in primary AECs with deficiency of the ion channel CF transmembrane conductance regulator (CFTR). Further, we show that the main component of *P. aeruginosa* flagella, the protein flagellin, increases TMPRSS2 expression in primary AECs and Calu-3 cells, through activation of Toll-like receptor-5 and p38 MAPK. This increase is particularly seen in Calu-3 cells deficient for CFTR and is associated with an intracellular increased level of SARS-CoV-2 infection, however, with no effect on the amount of virus particles released. Considering the urgency of the COVID-19 health crisis, this result may be of clinical significance for CF patients, who are frequently infected with and colonized by *P. aeruginosa* during the course of CF and might develop COVID-19.

## Introduction

As of October 30, 2021, the coronavirus disease 2019 (COVID-19) pandemic, caused by severe acute respiratory syndrome (SARS)-coronavirus (CoV)-2, has infected nearly 245 million people globally and led to >4.9 million deaths (https://covid19.who.int). In this health crisis, one of the major challenges is to identify the susceptibility factors of the infecting virus in order to adapt public health recommendations and to reduce the risk of getting COVID-19, particularly in the case of the most vulnerable people: patients with common chronic respiratory diseases such as asthma and chronic obstructive pulmonary disease, and patients with less common or rare chronic respiratory diseases such as cystic fibrosis (CF). Given their lung impairments, patients with chronic respiratory diseases can reasonably be expected to face an elevated risk of developing severe COVID-19, but the magnitude of this risk remains uncertain (1). Together with clinical follow-up studies conducted to more accurately estimate the disease risk of these patients, basic research on the pathophysiology of SARS-CoV-2 infection should provide critical insights into how COVID-19 affects patients with respiratory diseases.

The aforementioned COVID-19 development in patients is particularly relevant in the case of people with CF (pwCF). CF is caused by variants in the gene *CFTR* (CF transmembrane conductance regulator), with the most frequent variant being F508del, which leads to aberrant function of airway epithelial cells (AECs). During the course of CF, the lungs of the patients are inflamed and chronically infected by various pathogens, including *Pseudomonas aeruginosa*, the most prevalent pathogen (2). The most recent multinational report identified 181 cases of pwCF infected by SARS-CoV-2, and recorded 7 deaths (3); among the 181 pwCF, 82% were symptomatic, 47% were hospitalized, and 51% showed airway infection by *P. aeruginosa*.

AECs play a critical role in the regulation of both the immune response and the severity of COVID-19 (4). Notably, several studies examining SARS-CoV-2 cellular tropism have demonstrated that ciliated and secretory cells are the major targets of infection (4–8). SARS-CoV-2 infects the airway mainly through the cell-surface receptor angiotensin-converting enzyme-2 (ACE2), and two specific host proteases, TMPRSS2 (transmembrane serine protease 2) and FURIN, have been shown to play a major role in SARS-CoV-2 infectivity (9–13).

Here, we show that the main component of *P. aeruginosa* flagella, the protein flagellin (*Pa*-F), upregulates TMPRSS2 expression in AECs, especially in patients’ cells deficient for *CFTR*, through Toll-like receptor-5 (TLR5) and p38 activation. Importantly, this enhanced TMPRSS2 expression is associated with an increase in the level of SARS-CoV-2 infection.

## Material and Methods

### Reagents

Ultrapure flagellin from *P. aeruginosa* (tlrl-pafla) and ultrapure (tlrl-epstfla), recombinant (tlrl-flic), and vaccigrade (vac-fla) flagellin from *Salmonella enterica* serovar Typhimurium were from InvivoGen (San Diego, CA, USA). Anti-TLR5 antibody and NF-κB inhibitor (BAY 11-7082) were from InvivoGen. DMSO and p38 inhibitor (SB203580) were from Sigma-Aldrich (Saint-Louis, MO, USA).

### Cell Culture

Calu-3 cells (ATCC HTB-55™/Lot: 62657853) and Calu-3-*CFTR*-WT and Calu-3-*CFTR*-KD cells (generously provided by Prof. Marc Chanson, University of Geneva, Switzerland) were cultured in MEM-Glutamax (Gibco, Paisley, UK) supplemented with 10% fetal calf serum (FCS; Eurobio, Les Ulis, France) and 1% non-essential amino acids, 10 mmol/L HEPES (pH 7.2-7.5), 1% sodium pyruvate, and 1% antibiotics (all from Gibco). They grow at the air-liquid interface, in Transwell^®^ dishes (12 mm; 3460, Corning, Kennebunk, ME, USA), to obtain polarized cells as previously described (14). Primary human bronchial epithelial cells (source characteristics listed in **Table 1**) were cultured as recommended by the manufacturer by using hAEC complete culture medium (Epithelix, Geneva, Switzerland). Beas-2B cells (CRL-9609™/Lot: 59227035) were cultured in F12 medium supplemented with 10% FCS, 10 mmol/L HEPES, and 1% antibiotics. 16HBE14o- cells were generously supplied by Pr. Dieter Gruenert (originator) and Dr. Beate Illek (provider) from the University of California San Francisco (UCSF); the cells were cultured in MEM-Glutamax supplemented with 10% FCS and 1% antibiotics, as recommended by the provider. Caco-2/TC7 cell line, a clonal population established from human colon carcinoma Caco-2 cells at late passage (15), were generously provided by Dr. Véronique Carrière (Sorbonne Université/Centre de recherche St-Antoine); the cells were cultured in high-glucose DMEM-Glutamax (Gibco) supplemented with 20% FCS, 1% non-essential amino acids, and 1% antibiotics.

**Table 1:** Characteristics of donors of bronchial epithelial cells.

Group	Reference	Origin	Sex	Age	CFTR variant	Smoker	Used in
WT	02AB077201F2	Caucasian	Male	63	–	No	**Figure 1C**
	02AB068001F2	Caucasian	Female	71	–	No	**Figure 1C**
	02AB067101	Caucasian	Male	72	–	No	**Figure 1C**
	02AB0839.01	Caucasian	Male	54	–	No	**Figure 1C**
CF	CFAB043703	Unknown	Male	27	F508del/F508del	No	**Figure 1C**
	CFAB060901	Unknown	Female	21	F508del/F508del	No	**Figure 1C**
	CFAB045202	Unknown	Male	32	F508del/F508del	No	**Figure 1C**
	CFAB064901	Unknown	Female	37	F508del/1717-1G>A	No	**Figures 1C**, **3D, E**

### Reverse Transcription-qPCR

Human RNA was isolated using a NucleoSpin RNA/miRNA kit (Macherey Nagel, Duren, Germany). RT was performed using a high-capacity cDNA kit (Applied Biosystems, Foster City, CA, USA). Real-time qPCR was performed by using an ABI QS3 with a Sensifast Probe Lo-Rox Kit (Bio-technofix, Guibeville, France), TaqMan probes for *ACE2* (Hs01085333_m1), *TMPRSS2* (Hs00237175_m1), *FURIN* (Hs00965485_g1), and *GAPDH* (Hs02786624_g1), and a cDNA template. For relative quantification, the expression level of target genes was normalized to the expression of *GAPDH* relative to the reference group (specified in the figure legends) used as a calibrator and was calculated using the 2^−ΔΔCt^ method.

### SARS-CoV-2 Infection and Viral Quantification

Fully polarized Calu-3 cells grown at the air-liquid interface were infected with SARS-CoV-2 (strain BetaCoV/France/IDF0571/2020; accession ID EPI_ISL_411218) at a multiplicity of infection of 1, as previously described (1 h of contact with the virus followed by a change of the medium, and analysis at 24h) (16). Viral quantification through RT-qPCR targeting of ORF1b-nsp14 was performed as described (16).

### Western Blotting

Total proteins were extracted using RIPA buffer (Euromedex, Souffelweyersheim, France), and then equal amounts of proteins were reduced, size-separated on 12% stain-free precast SDS-polyacrylamide gels (Bio-Rad, Hercules, CA, USA), and transferred to nitrocellulose membranes by using an iBlot2 apparatus (Thermo Fisher Scientific). The membranes were blocked in 5% milk in TBS-Tween 0.1% and incubated with specific primary antibodies overnight at 4°C; the antibodies were against ACE2 (AF933, R&D Systems, Minneapolis, MN, USA; 1:200), phospho- and total p38 (9211 and 9212, Cell Signaling Technology, Danvers, MA, USA; 1:2,000), phospho- and total NF-κB p65 (3039 and 8242, Cell Signaling Technology; 1:2,000), and β-actin (A2228, Sigma-Aldrich; 1:5,000). The blots were exposed to horseradish peroxidase-conjugated anti-rabbit (Cell Signaling Technology, 7074; 1:10,000) and anti-goat (A27104, Thermo Fisher Scientific; 1:2,000) secondary antibodies, and bound antibodies were detected using Clarity chemiluminescent substrate (Bio-Rad). Images were recorded using a Fujifilm LAS-3000 bioimaging system (Stamford, CT, USA).

### Immunofluorescence

After various treatments, Calu-3 grown at the air-liquid interface were rinsed with PBS and fixed with ice-cold 4% paraformaldehyde for 20 min, permeabilized for 10 min with 0.1% Triton X-100 in PBS, and then washed with PBS and incubated in a blocking solution (PBS + 5% BSA) for 1 h. Next, the cells were incubated overnight at 4°C with primary antibodies against TMPRSS2 (14437-1-AP, Thermo Fisher Scientific; 1:100) or ACE2 (AF933, R&D Systems; 1:60) in PBS supplemented with 1% BSA, and on the following day, the cells were washed (3 × 5 min) with PBS and incubated for 1 h at room temperature with secondary antibodies, anti-rabbit Alexa 488 (4412, Cell Signaling Technology, 1:2,000) or anti-goat Alexa 488 (A11078, Thermo Fisher Scientific, 1:2,000). After staining with 4,6-diamidino-2-phenylindole (DAPI), coverslips were mounted and sealed with ProLong diamond mounting medium (Thermo Fisher Scientific). Fluorescent images were obtained using an Olympus BX43 microscope (Hamburg, Germany).

### ELISA

Concentrations of human IL-8, IL-6, IFN-β, IFN-λ in cell supernatants were measured using ELISA kits (DY208, DY206, DY814 and DY1598B, R&D Systems), according to the manufacturer’s instructions. The substrate 3,3′,5,5′-tetramethylbenzidine was from Cell Signaling Technology.

### Statistical Analysis

Differences among groups were assessed for statistical significance by using Prism 9.00 software (GraphPad Software, La Jolla, CA, USA), as indicated in the figure legends. *P* < 0.05 was considered statistically significant.

### Ethics

This project was approved (Opinion number 20-688) by the Inserm Institutional Review Board (IRB00003888, IORG0003254, FWA00005831).

### Data Availability

RNAseq data used here are from a transcriptomic study (17) which RNAseq raw datafiles are available in the European Nucleotide Archive (ENA) (primary accession number PRJEB9292). http://www.ebi.ac.uk/ena/data/view/PRJEB9292.

## Results

### *ACE2*, *FURIN*, and *TMPRSS2* Expression In CF and Non-CF Primary Human AECs Upon *P. aeruginosa* Infection

**Figure 1:**
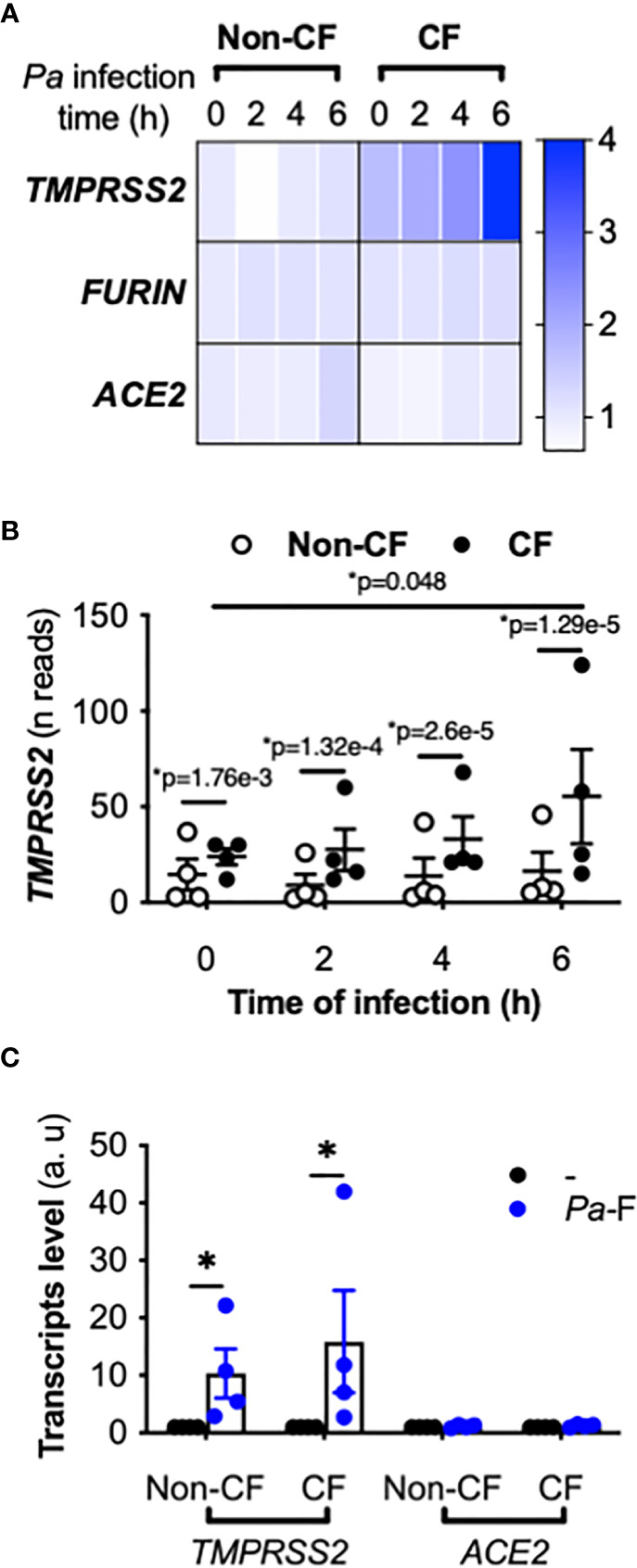
Effect of *P. aeruginosa* infection on *ACE2*, *TMPRSS2*, and *FURIN* expression in primary hAECs. Heatmap of *ACE2*, *TMPRSS2*, and *FURIN* expression (fold-change) **(A)** and kinetics of *TMPRSS2* expression (shown in reads) **(B)** in primary hAECs isolated from non-CF and CF patients and infected with *P. aeruginosa* (multiplicity of infection = 0.25) [RNA-seq data extracted from a previous study (17), Benjamini-Hochberg adjusted p value (*p)]. **(C)** *ACE2* and *TMPRSS2* mRNA expression (in arbitrary units, a.u.) in submerged non-CF (n=4) and CF (n=4) primary hAECs stimulated with control medium (reference group) or *Pa*-F (50 ng/mL) for 6 h (ANOVA with Bonferroni’s multiple-comparison test, *P < 0.05).

We first examined *ACE2*, *FURIN*, and *TMPRSS2* expression from a previous transcriptomic study performed using primary hAECs; the cells were isolated from control (non-CF) donors and pwCF homozygous for the *CFTR* F508del variant, and were infected by *P. aeruginosa* (17). At baseline (time 0 h), similar *ACE2* and *FURIN* mRNA expression levels were observed in non-CF and CF primary hAECs (**Figure 1A**), whereas *TMPRSS2* expression was significantly higher in CF primary hAECs (**Figures 1A, B**). Importantly, *P. aeruginosa* infection increased *TMPRSS2* mRNA expression over time in CF but not in non-CF primary hAECs (**Figures 1A, B**), whereas the infection did not affect *ACE2* and *FURIN* expression (**Figure 1A**).

Because the most critical proinflammatory factor from *P. aeruginosa* present in the sputum of pwCF is flagellin (18), we next exposed CF primary hAECs to flagellin. Treatment with flagellin increased the mRNA level of *TMPRSS2* without increasing that of *ACE2* (**Figure 1C**) or *FURIN* (not illustrated). This effect was observed in both non-CF and CF primary hAECs, although in both groups, the level of induction varied considerably between individuals.

### *ACE2*, *FURIN*, and *TMPRSS2* Expression in *CFTR*-Sufficient and -Deficient Calu-3 Cells Exposed to *P. aeruginosa Flagellin*

**Figure 2:**
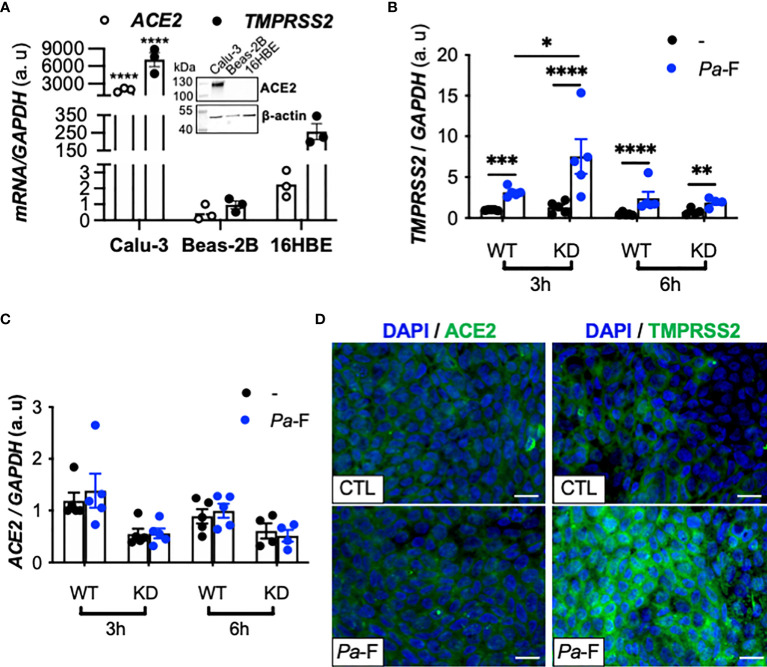
Effect of *P. aeruginosa* flagellin on ACE2, FURIN, and TMPRSS2 expression in *CFTR*-deficient Calu-3 cells. **(A)** *ACE2* and *TMPRSS2* mRNA expression in submerged cultures of Calu-3, Beas-2B (reference), and 16HBE14o- cell lines (*n* = 3, ANOVA with Dunnett’s multiple-comparison test, control group: Calu-3, ****P < 0.0001). *GAPDH*, housekeeping gene. Representative western blot (with 20 μg of protein) showing ACE2 and β-actin protein expression in submerged cultures of Calu-3, Beas-2B, and 16HBE14o- cell lines. *TMPRSS2* **(B)** and *ACE2* **(C)** mRNA expression (relative to that of housekeeping gene *GAPDH*) in Calu-3-*CFTR*-WT (reference group) and -*CFTR*-KD cells grown at the air-liquid interface and either not stimulated (–) or stimulated for 3 or 6 h with *P. aeruginosa* flagellin (*Pa*-F, 50 ng/mL) (*n* = 5, ANOVA with Bonferroni’s multiple-comparison test, *P < 0.05, **P < 0.01, ***P < 0.001, ****P < 0.0001). **(D)** Immunofluorescence analysis of TMPRSS2 and ACE2 protein expression in Calu-3 cells (ATCC) grown at the air-liquid interface and stimulated with *Pa*-F for 18 h; scale bar, 20 μm.

To investigate the mechanism underlying the aforementioned increase in TMPRSS2 expression and to eliminate the interindividual variability, we sought to identify AEC lines expressing detectable levels of ACE2 and TMPRSS2 mRNA and protein. Thus, we measured ACE2 and TMPRSS2 expression in the AEC lines Calu-3, Beas-2B, and 16HBE (**Figure 2A**), which revealed that Calu-3 cells expressed higher mRNA levels of *ACE2* and *TMPRSS2* relative to the other cell lines, and that ACE2 protein was detected only in Calu-3 cells. These results agree with the documented higher ability of SARS-CoV-2 to replicate in Calu-3 cells than in Beas-2B cells (10). Thus, we hereafter used the Calu-3 cell line, specifically isogenic *CFTR*-sufficient (Calu-3-*CFTR*-WT) and *CFTR*-deficient Calu-3 (Calu-3-*CFTR*-KD) cells.

In accord with what was observed in CF primary hAECs, we found that exposure of Calu-3 cells to *P. aeruginosa* flagellin significantly increased *TMPRSS2* mRNA expression (**Figure 2B**) in a dose-dependent manner (**Supplementary Figure 1**) without affecting the transcripts levels of *ACE2* (**Figure 2C**) and *FURIN* (not illustrated). This increase in *TMPRSS2* expression was more notable in Calu-3-*CFTR*-KD than in Calu-3-*CFTR*-WT (**Figure 2B**), and the TMPRSS2 upregulation was also detected at the protein level (**Figure 2D**). As expected, flagellin induced the synthesis of the proinflammatory cytokines interleukin (IL)-8 and IL-6 (**Supplementary Figure 2A, B**) both in Calu-3-*CFTR*-WT and Calu-3-*CFTR*-KD cells, and this inflammatory response was relatively higher in the Calu-3-*CFTR*-KD cells, which agrees with previous work showing that CF epithelial cells from human (19) or porcine (20) origin exhibit an enhanced inflammatory response to flagellin.

**Figure 3:**
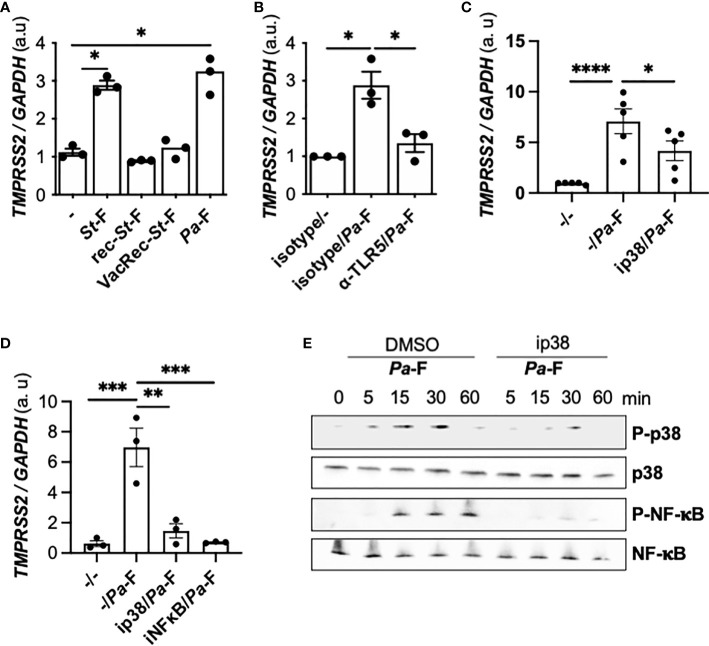
Effect of TLR5 and p38 inhibition on flagellin induced-TMPRSS2 expression in *CFTR*-deficient cells. **(A)** *TMPRSS2* mRNA expression in Calu-3-*CFTR*-KD grown at the air-liquid interface and either not stimulated or stimulated for 6 h with 50 ng/mL ultrapure flagellin from S. Typhimurium (*St*)-F, recombinant *St*-F, vaccigrade *St*-*F*, or *Pa*-F (*n* = 3, ANOVA with Dunnett’s multiple-comparison test, *P < 0.05). **(B)** *TMPRSS2* mRNA expression in Calu-3-*CFTR*-KD cells grown at the air-liquid interface and incubated for 1 h with isotype control (reference) or anti-TLR5 antibody (10 μg/mL) and then either not stimulated or stimulated for 6 h with *Pa*-F (50 ng/mL) (*n* = 3, ANOVA with Dunnett’s multiple-comparison test, control group: Isotype/*Pa*-F, *P < 0.05). *TMPRSS2* mRNA expression in Calu-3-*CFTR*-KD cells grown at the air-liquid interface **(C)** or in primary CF hAECs **(D)** that were preincubated for 1 h with 20 μmol/L p38 or 20 μmol/L NF-κB inhibitors and then stimulated for 6 h with 50 ng/mL *Pa*-F in the presence of the inhibitor. ANOVA with Dunnett’s multiple-comparison test, control group: -/*Pa*-F, *P < 0.05, **P < 0.01, ***P < 0.001, ****P < 0.0001. **(E)** Western blot (with 10 μg of protein) of phospho- and total p38 and phospho- and total NF-κB in primary hAECs stimulated with 50 ng/mL *Pa*-F.

To ascertain whether the observed effect of flagellin is specific to the bacterial source of the protein, we used ultrapure flagellin isolated from *S. Typhimurium* serovar Typhimurium (*S*t*-*F) in our assays, which revealed that *St*-F induced similar *TMPRSS2* expression in Calu-3 cells as did flagellin isolated from *P. aeruginosa* (**Figure 3A**). By contrast, recombinant flagellins (standard or vaccigrade™) from *S.* Typhimurium did not affect the expression (**Figure 3A**).

Flagellin is known to activate TLR5 and the downstream p38 mitogen-activated protein kinase (MAPK) signaling pathway (21); thus, we tested the involvement of this pathway in the observed *TMPRSS2* induction. We showed that the upregulation of *TMPRSS2* expression depended on TLR5 signaling (**Figure 3B**). When a p38 inhibitor was used, flagellin-induced *TMPRSS2* expression was diminished in Calu-3-*CFTR*-KD cells (**Figure 3C**) and primary CF hAECs (**Figure 3D**). Furthermore, an inhibitor of nuclear factor-kappa B (NF-κB) also reduced the *TMPRSS2* induction by flagellin. Accordingly, the results of western blotting confirmed that flagellin stimulated p38 phosphorylation as well as NF-κB activation (**Figure 3E**). Moreover, as shown previously (21), we further observed that this NF-κB activation depended on p38 activity (**Figure 3E**).

To determine whether *TMPRSS2* induction was restricted to the lung epithelium, we examined the flagellin effect in an intestinal cell line, Caco-2/TC7. Our results showed that flagellins from *P. aeruginosa* and S. Typhimurium, which were able to induce IL-8 production (**Supplementary Figure 3A**), exerted no effect on *TMPRSS2* expression in these cells (**Supplementary Figure 3B**).

### Influence of TMPRSS2 Induction by *P. aeruginosa* Flagellin on SARS-CoV-2 Infectivity in *CFTR*-Sufficient and -Deficient Calu-3 Cells

**Figure 4:**
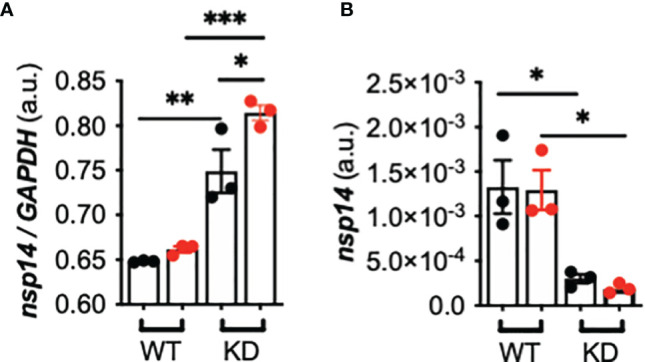
Effect of TMPRSS2 induction by *P. aeruginosa* flagellin on SARS-CoV-2 infectivity in *CFTR*-deficient Calu-3 cells. **(A)** Intracellular *nsp14/GAPDH* and **(B)** apical (supernatant) *nsp14* mRNA expression in Calu-3-*CFTR*-WT (reference group) and -*CFTR*-KD cells grown at the air-liquid interface and either not stimulated (black circle) or stimulated (red circle) for 16 h with *Pa*-F 50 ng/mL), and then infected for 24 h with SARS-CoV-2 (multiplicity of infection = 1) (*n* = 3, ANOVA with Bonferroni’s multiple-comparison test, *P < 0.05, **P < 0.01, ***P < 0.001).

Lastly, we investigated whether TMPRSS2 induction by flagellin influences SARS-CoV-2 infectivity. After infection with SARS-CoV-2, the intracellular *nsp14* viral mRNA level was increased, and this level was significantly higher in Calu-3-*CFTR*-KD than -*CFTR*-WT cells and was even more notably elevated when the cells were pre-stimulated with flagellin (**Figure 4A**). By contrast, the extracellular *nsp14* viral mRNA level, measured in the apical supernatant of AECs as a surrogate for viral production, was significantly lower in Calu-3-*CFTR*-KD cells than in Calu-3-*CFTR*-WT cells (**Figure 4B**). Whereas pre-stimulation with flagellin did not affect viral-particle release in Calu-3-*CFTR*-WT cells, a lower, albeit not statistically significant, level of *nsp14* mRNA was measured at the apical side of Calu-3-*CFTR*-KD cells pre-stimulated with flagellin. To ensure that viral particles release was not due to an increase in the permeability of the epithelial barrier by flagellin, we measured TEER and observed that flagellin treatment was not associated with a loss of epithelial integrity (**Supplementary Figure 4**). Lastly, IFN-β and IFN-λ measurements in basal supernatants were not induced either by the virus or the flagellin in the different conditions tested (not illustrated).

## Discussion

**Figure 5:**
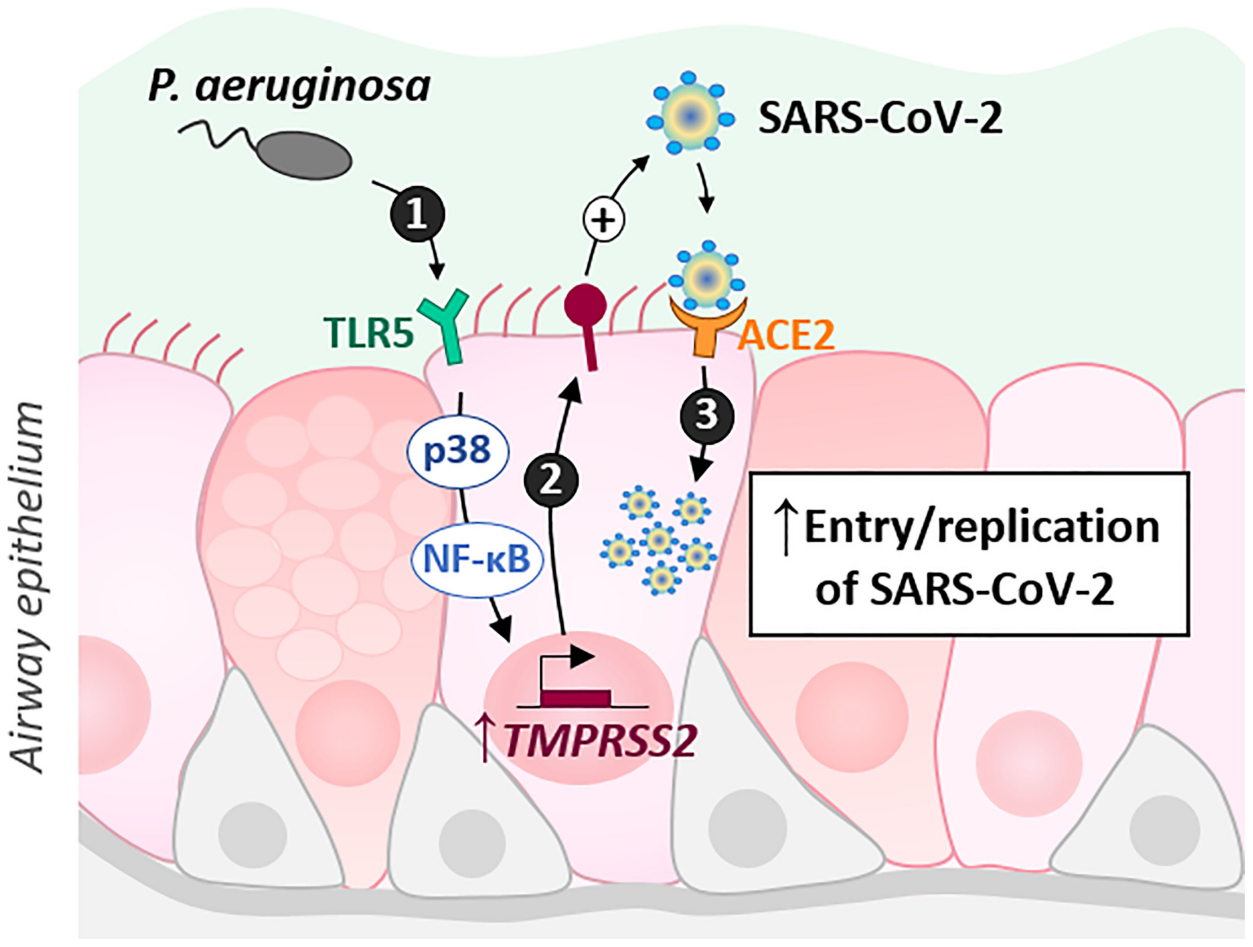
Schematic illustrating the results. *P. aeruginosa* interacts with the airway epithelial cells of CF patients, notably by activating the TLR5 signaling pathway through its virulence factor, flagellin. This activation, dependent on p38 MAPK and NF-kB, leads to an increase in TMPRSS2 which could regulate SARS-CoV2 infectivity.

In this study, we showed that exposure of AECs to flagellin from *P. aeruginosa* induces an increase in TMPRSS2 expression, which is dependent on TLR5 and p38 MAPK activation. Notably, prior exposure of AECs to flagellin results in increased infectivity of SARS-CoV-2 (illustrated in **Figure 5**).

We found that *TMPRSS2* is more expressed in hAECs from pwCF as compared with the level in controls. Although the hAECs were isolated from a limited number of pwCF, this observation agrees with previous results obtained using excised lungs, where RNA *in situ* hybridization revealed that TMPRSS2 expression was higher in pwCF than in non-CF patients (7). We also observed that flagellin from *P. aeruginosa* increases TMPRSS2 expression in primary bronchial epithelial cells from both pwCF and controls. The variability of the flagellin-elicited response in the two groups is likely due to the effect of several donor-related factors, such as sex, age, and CF clinical history in the case of pwCF. An age-related increase in TLR5 expression and sensing has been observed in human monocytes (22). Moreover, the single-nucleotide polymorphism *TLR5* c.1174C>T, which is common in the general population (23) and generates a variant that acts as a modifier gene in CF (24), might also contribute to this variability. Unfortunately, it was not possible to have age-matched donors which constitutes a limitation in our study.

To elucidate the specific contribution of CFTR in the level of TMPRSS2 induction by flagellin, we used isogenic Calu-3-*CFTR*-WT and Calu-3-*CFTR*-KD cells. We found that TMPRSS2 is highly induced in Calu-3-*CFTR*-KD cells, and we further confirmed previous observations indicating that *CFTR*-deficient bronchial cells show an elevated inflammatory response to flagellin, characterized by increased levels of IL-6 and IL-8 (19). Flagellin is known to activate TLR5 and downstream p38 and NF-κB in Calu-3 cells (21). Confirming these results, TMPRSS2 induction by flagellin was found here to depend on p38 and NF-κB, both in Calu-3 cells and primary CF hAECs. Interestingly, a recent phosphoproteomic study in Vero-6 cells revealed that SARS-CoV-2 stimulates the p38 pathway, and that pharmacological inhibition of p38 shows antiviral efficacy (25). Thus, this study and our results here suggest that inhibiting the p38 pathway could represent a potential COVID-19 therapy.

The increase in intracellular viral mRNA levels in CF cells exposed to flagellin, which indicates an elevated level of infection, is likely the result of the upregulated expression of TMPRSS2. Accordingly, TMPRSS2 inhibition by using the serine-protease inhibitor camostat mesylate is sufficient for preventing infection with SARS-CoV-2 (10). Intriguingly, we observed a lower level of viral particles at the apical side of CF cells as compared with the levels in non-CF cells; this could be the result of an increased host-defense capacity of CF cells against viral infection, or a delay in the kinetics of virus release. Interestingly, a recent transcriptomic study shows that despite the fact that influenza virus (IAV) replication in Calu3-*CFTR*-WT and Calu-3-*CFTR*-KD cells is similar, a specific immune gene profile is observed in Calu-3-*CFTR*-KD before and after infection with IAV. It is also shown by RNAseq analysis that flagellin stimulation revealed potential dysregulation of pathways involved in viral infection. Importantly, by stimulating cells with IFNs and using a transcription network analysis, they also demonstrated that IFN signaling is altered in *CFTR* deficient cells (26) which could explain in part our results. Future studies specifically investigating infection kinetics, viral-particle release, and the resulting antiviral response including the production of IL-6 and Type I/III interferons should facilitate definitive assessment of whether prior exposure to flagellin induces either a protective or damaging effect after SARS-CoV-2 infection of CF cells. The non-detection of IFN-β and IFN-λ in our viral infection conditions is probably related to the very short infection time chosen, which if it allows to study the initiation of the viral infection process does not allow to study at best the resulting inflammatory response. Indeed, it was shown in Calu-3 cells that SARS-CoV-2 induces a significant but delayed IFN-β in comparison to Sendaï virus or synthetic dsRNA (poly I:C) (27).

Further investigations conducted using primary cells differentiated at the air-liquid interface will be necessary to specifically characterize the response of CF bronchial epithelial cells. Nevertheless, the model used here is relevant. As noted in the introduction section, secretory cells are infected by SARS-CoV-2; this was demonstrated in previous studies conducted using single-cell RNA-seq, either ex vivo with lung biopsies of patients infected with SARS-CoV-2 (4), or *in vitro* with a reconstituted epithelium at the air-liquid interface (6, 8). Calu-3 cells polarized at the air-liquid interface present the characteristics of secretory cells (mucus production) and express naturally (i.e., without exogenous overexpression) the required proteins (ACE2, TMPRSS2) for infection by SARS-CoV-2, which is not the case with the other epithelial cell lines tested in this study.

Several studies have delineated the antiviral capacity endowed by flagellin against other respiratory viruses, including influenza A (28). Furthermore, flagellin was recently suggested to be capable of modulating the innate immune response and thereby eliminating SARS-CoV-2 and resolving COVID-19 (29). Accordingly, the use of recombinant flagellin as an adjuvant in vaccine development has been considered (30). However, we observed here that recombinant flagellin (standard or vaccigrade™) from *S.* Typhimurium did not affect *TMPRSS2* expression. Thus, although these data were obtained *in vitro*, it is likely based on the aforementioned finding that pathogen-targeting vaccines developed using recombinant flagellin as an adjuvant will not produce negative effects in the case of concomitant exposure to SARS-CoV-2. Moreover, the observed upregulation of TMPRSS2 expression induced by flagellin appears specific to the lung epithelium, because the effect was not replicated in Caco-2/TC7 cells, which are epithelial cells of intestinal origin. During their lifetime, pwCF are also infected with other flagellated bacteria including *Burkholderia cenocepacia* and *Stenotrophomonas maltophilia* (31). In particular, flagellin from *Burkholderia cenocepacia* also known to activate TLR5 (32, 33), could also modulate TMPRSS2 expression.

The question of whether CF patients face an increased risk of developing a severe form of COVID-19 is a topical one and a source of discussion (34). Clinical follow-up results obtained to date indicate that pwCF, both adults (3, 35) and children (36), do not show an elevated risk of developing severe COVID-19 as compared with the general population. However, pwCF with advanced CF disease (associated with older age, CF-related diabetes, lower lung function, having received an organ transplant) might develop a severe clinical course (3, 35).

In a recent French study, we compared the baseline clinical characteristics of 31 pwCF infected by SARS-CoV-2 during the first wave of the pandemic to that of the overall French CF population (*n* = 6,913; >90% of all French CF cases) (37). The pwCF with COVID-19 were found to be older and more frequently chronically colonized with *P. aeruginosa*  *(* 37). However, considering the small number of patients, these results must be interpreted with caution. Thus, whether the risk of developing severe COVID-19 is increased in pwCF because of their *P. aeruginosa* infection remains unresolved and will require further meta-analysis performed using international cohorts. Moreover, a recent study including 874 individuals with COVID-19 showed that carriers of CF-causing variants (N=40) may be more likely to develop severe COVID-19 (38). This reinforces the interest in studying the specific role of CFTR in the pathogenesis of COVID-19.

In conclusion, we have shown that exposure of CF AECs to flagellin from *P. aeruginosa* can enhance SARS-CoV-2 infectivity. Further clinical follow-up studies and *in vitro* experimental investigations into the mechanisms associated with the specific host response of primary CF cells to SARS-CoV-2 infection should help elucidate this matter and provide insights for future clinical care.

## Data Availability Statement

The datasets presented in this study can be found in online repositories. The names of the repository/repositories and accession number(s) can be found below: https://www.ebi.ac.uk/ena, PRJEB9292.

## Author Contributions

LG, MR, and OT, designed experiments. MR, JB, CC, VB, OT, AP, JM, MG, JO, conducted the experiments. LG wrote the manuscript. MR, JB, VB, MR-C, HC and OT critically revised the manuscript. All authors contributed to the article and approved the submitted version.

## Funding

LG received a grant from the Faculté de Médecine Sorbonne Université (AAP COVID19).

## Conflict of Interest

The authors declare that the research was conducted in the absence of any commercial or financial relationships that could be construed as a potential conflict of interest.

## Publisher’s Note

All claims expressed in this article are solely those of the authors and do not necessarily represent those of their affiliated organizations, or those of the publisher, the editors and the reviewers. Any product that may be evaluated in this article, or claim that may be made by its manufacturer, is not guaranteed or endorsed by the publisher.

## References

[1] To T Viegi G Cruz A Taborda-Barata L Asher M Behera D. A Global Respiratory Perspective on the COVID-19 Pandemic: Commentary and Action Proposals. Eur Respir J (2020) 56(1):2001704. doi: 10.1183/13993003.01704-2020 32586874 PMC7315811

[2] Elborn JS. Cystic Fibrosis. Lancet (2016) 388(10059):2519–31. doi: 10.1016/S0140-6736(16)00576-6 27140670

[3] McClenaghan E Cosgriff R Brownlee K Ahern S Burgel PR Byrnes CA. The Global Impact of SARS-CoV-2 in 181 People With Cystic Fibrosis. J Cyst Fibros (2020) 19(6):868–71. doi: 10.1016/j.jcf.2020.10.003 PMC7641525 33183965

[4] Chua RL Lukassen S Trump S Hennig BP Wendisch D Pott F. COVID-19 Severity Correlates With Airway Epithelium-Immune Cell Interactions Identified by Single-Cell Analysis. Nat Biotechnol (2020) 38(8):970–9. doi: 10.1038/s41587-020-0602-4 32591762

[5] Zhu N Wang W Liu Z Liang C Wang W Ye F. Morphogenesis and Cytopathic Effect of SARS-CoV-2 Infection in Human Airway Epithelial Cells. Nat Commun (2020) 11(1):3910. doi: 10.1038/s41467-020-17796-z 32764693 PMC7413383

[6] Ravindra NG Alfajaro MM Gasque V Huston NC Wan H Szigeti-Buck K. Single-Cell Longitudinal Analysis of SARS-CoV-2 Infection in Human Airway Epithelium Identifies Target Cells, Alterations in Gene Expression, and Cell State Changes. PloS Biol (2021) 19(3):e3001143. doi: 10.1371/journal.pbio.3001143 33730024 PMC8007021

[7] Hou YJ Okuda K Edwards CE Martinez DR Asakura T Dinnon KH 3rd. SARS-CoV-2 Reverse Genetics Reveals a Variable Infection Gradient in the Respiratory Tract. Cell (2020) 182(2):429–446 e14. doi: 10.1016/j.cell.2020.05.042 32526206 PMC7250779

[8] Fiege JK Thiede JM Nanda HA Matchett WE Moore PJ Montanari NR. Single Cell Resolution of SARS-CoV-2 Tropism, Antiviral Responses, and Susceptibility to Therapies in Primary Human Airway Epithelium. PloS Pathog (2021) 17(1):e1009292. doi: 10.1371/journal.ppat.1009292 33507952 PMC7872261

[9] Coutard B Valle C de Lamballerie X Canard B Seidah NG Decroly E. The Spike Glycoprotein of the New Coronavirus 2019-Ncov Contains a Furin-Like Cleavage Site Absent in CoV of the Same Clade. Antiviral Res (2020) 176:104742. doi: 10.1016/j.antiviral.2020.104742 32057769 PMC7114094

[10] Hoffmann M Kleine-Weber H Schroeder S Kruger N Herrler T Erichsen S. SARS-CoV-2 Cell Entry Depends on ACE2 and TMPRSS2 and Is Blocked by a Clinically Proven Protease Inhibitor. Cell (2020) 181(2):271–80.e8. doi: 10.1016/j.cell.2020.02.052 PMC7102627 32142651

[11] Walls AC Park YJ Tortorici MA Wall A McGuire AT Veesler D. Structure, Function, and Antigenicity of the SARS-CoV-2 Spike Glycoprotein. Cell (2020) 183(6):1735. doi: 10.1101/2020.02.19.956581 33306958 PMC7833104

[12] Yan R Zhang Y Li Y Xia L Guo Y Zhou Q. Structural Basis for the Recognition of the SARS-CoV-2 by Full-Length Human ACE2. Science (2020) 367(6485):1444–8. doi: 10.1101/2020.02.19.956946 PMC7164635 32132184

[13] Zhou P Yang XL Wang XG Hu B Zhang L Zhang W. A Pneumonia Outbreak Associated With a New Coronavirus of Probable Bat Origin. Nature (2020) 579(7798):270–3. doi: 10.1038/s41586-020-2012-7 PMC7095418 32015507

[14] Bellec J Bacchetta M Losa D Anegon I Chanson M Nguyen TH. CFTR Inactivation by Lentiviral Vector-Mediated RNA Interference and CRISPR-Cas9 Genome Editing in Human Airway Epithelial Cells. Curr Gene Ther (2015) 15(5):447–59. doi: 10.2174/1566523215666150812115939 26264708

[15] Chantret I Rodolosse A Barbat A Dussaulx E Brot-Laroche E Zweibaum A. Differential Expression of Sucrase-Isomaltase in Clones Isolated From Early and Late Passages of the Cell Line Caco-2: Evidence for Glucose-Dependent Negative Regulation. J Cell Sci (1994) 107( Pt 1):213–25. doi: 10.1242/jcs.107.1.213 8175910

[16] Pizzorno A Padey B Julien T Trouillet-Assant S Traversier A Errazuriz-Cerda E. Characterization and Treatment of SARS-CoV-2 in Nasal and Bronchial Human Airway Epithelia. Cell Rep Med (2020) 1(4):100059. doi: 10.1016/j.xcrm.2020.100059 32835306 PMC7373044

[17] Balloy V Varet H Dillies MA Proux C Jagla B Coppee JY. Normal and Cystic Fibrosis Human Bronchial Epithelial Cells Infected With Pseudomonas Aeruginosa Exhibit Distinct Gene Activation Patterns. PloS One (2015) 10(10):e0140979. doi: 10.1371/journal.pone.0140979 26485688 PMC4618526

[18] Balloy V Thevenot G Bienvenu T Morand P Corvol H Clement A. Flagellin Concentrations in Expectorations From Cystic Fibrosis Patients. BMC Pulm Med (2014) 14:100. doi: 10.1186/1471-2466-14-100 24909229 PMC4060841

[19] Blohmke CJ Victor RE Hirschfeld AF Elias IM Hancock DG Lane CR. Innate Immunity Mediated by TLR5 as a Novel Antiinflammatory Target for Cystic Fibrosis Lung Disease. J Immunol (2008) 180(11):7764–73. doi: 10.4049/jimmunol.180.11.7764 18490781

[20] Fleurot I Lopez-Galvez R Barbry P Guillon A Si-Tahar M Bahr A. TLR5 Signalling is Hyper-Responsive in Porcine Cystic Fibrosis Airways Epithelium. J Cyst Fibros (2021). doi: 10.1016/j.jcf.2021.08.002 34420900

[21] Illek B Fu Z Schwarzer C Banzon T Jalickee S Miller SS. Flagellin-Stimulated Cl- Secretion and Innate Immune Responses in Airway Epithelia: Role for P38. Am J Physiol Lung Cell Mol Physiol (2008) 295(4):L531–42. doi: 10.1152/ajplung.90292.2008 PMC2575945 18658272

[22] Qian F Wang X Zhang L Chen S Piecychna M Allore H. Age-Associated Elevation in TLR5 Leads to Increased Inflammatory Responses in the Elderly. Aging Cell (2012) 11(1):104–10. doi: 10.1111/j.1474-9726.2011.00759.x PMC3257374 22023165

[23] Hawn TR Verbon A Lettinga KD Zhao LP Li SS Laws RJ. A Common Dominant TLR5 Stop Codon Polymorphism Abolishes Flagellin Signaling and is Associated With Susceptibility to Legionnaires' Disease. J Exp Med (2003) 198(10):1563–72. doi: 10.1084/jem.20031220 PMC2194120 14623910

[24] Blohmke CJ Park J Hirschfeld AF Victor RE Schneiderman J Stefanowicz D. TLR5 as an Anti-Inflammatory Target and Modifier Gene in Cystic Fibrosis. J Immunol (2010) 185(12):7731–8. doi: 10.4049/jimmunol.1001513 21068401

[25] Bouhaddou M Memon D Meyer B White KM Rezelj VV Correa Marrero M. The Global Phosphorylation Landscape of SARS-CoV-2 Infection. Cell (2020) 182(3):685–712 e19. doi: 10.1016/j.cell.2020.06.034 32645325 PMC7321036

[26] Sofoluwe A Zoso A Bacchetta M Lemeille S Chanson M. Immune Response of Polarized Cystic Fibrosis Airway Epithelial Cells Infected With Influenza A Virus. J Cyst Fibros (2020) 20(4):655–63. doi: 10.1016/j.jcf.2020.08.012 32873524

[27] Lei X Dong X Ma R Wang W Xiao X Tian Z. Activation and Evasion of Type I Interferon Responses by SARS-CoV-2. Nat Commun (2020) 11(1):3810. doi: 10.1038/s41467-020-17665-9 32733001 PMC7392898

[28] Georgel AF Cayet D Pizzorno A Rosa-Calatrava M Paget C Sencio V. Toll-Like Receptor 5 Agonist Flagellin Reduces Influenza A Virus Replication Independently of Type I Interferon and Interleukin 22 and Improves Antiviral Efficacy of Oseltamivir. Antiviral Res (2019) 168:28–35. doi: 10.1016/j.antiviral.2019.05.002 31078648

[29] Golonka RM Saha P Yeoh BS Chattopadhyay S Gewirtz AT Joe B. Harnessing Innate Immunity to Eliminate SARS-CoV-2 and Ameliorate COVID-19 Disease. Physiol Genomics (2020) 52(5):217–21. doi: 10.1152/physiolgenomics.00033.2020 PMC7200864 32275178

[30] Cui B Liu X Fang Y Zhou P Zhang Y Wang Y. Flagellin as a Vaccine Adjuvant. Expert Rev Vaccines (2018) 17(4):335–49. doi: 10.1080/14760584.2018.1457443 29580106

[31] Blanchard AC Waters VJ. Microbiology of Cystic Fibrosis Airway Disease. Semin Respir Crit Care Med (2019) 40(6):727–36. doi: 10.1055/s-0039-1698464 PMC7117079 31887768

[32] Urban TA Griffith A Torok AM Smolkin ME Burns JL Goldberg JB. Contribution of Burkholderia Cenocepacia Flagella to Infectivity and Inflammation. Infect Immun (2004) 72(9):5126–34. doi: 10.1128/IAI.72.9.5126-5134.2004 PMC517433 15322006

[33] de CVGM Le Goffic R Balloy V Plotkowski MC Chignard M Si-Tahar M. TLR 5, But Neither TLR2 Nor TLR4, is Involved in Lung Epithelial Cell Response to Burkholderia Cenocepacia. FEMS Immunol Med Microbiol (2008) 54(1):37–44. doi: 10.1111/j.1574-695X.2008.00453.x 18680520

[34] Peckham D McDermott MF Savic S Mehta A. COVID-19 Meets Cystic Fibrosis: For Better or Worse? Genes Immun (2020) 21(4):260–2. doi: 10.1038/s41435-020-0103-y 32606316

[35] Cosgriff R Ahern S Bell SC Brownlee K Burgel PR Byrnes C. A Multinational Report to Characterise SARS-CoV-2 Infection in People With Cystic Fibrosis. J Cyst Fibros (2020) 19(3):355–8. doi: 10.1016/j.jcf.2020.04.012 PMC7183287 32376098

[36] Bain R Cosgriff R Zampoli M Elbert A Burgel PR Carr SB. Clinical Characteristics of SARS-CoV-2 Infection in Children With Cystic Fibrosis: An International Observational Study. J Cyst Fibros (2021) 20(1):25–30. doi: 10.1016/j.jcf.2020.11.021 33309057 PMC7713571

[37] Corvol H de Miranda S Lemonnier L Kemgang A Reynaud Gaubert M Chiron R. First Wave of COVID-19 in French Patients With Cystic Fibrosis. J Clin Med (2020) 9(11). doi: 10.3390/jcm9113624 PMC7697588 33182847

[38] Baldassarri M Fava F Fallerini C Daga S Benetti E Zguro K. Severe COVID-19 in Hospitalized Carriers of Single CFTR Pathogenic Variants. J Pers Med (2021) 11(6). doi: 10.3390/jpm11060558 PMC8232773 34203982

[39] Ruffin M Bigot J Calmel C Mercier J Pizzorno A Rosa-Calatrava M. Flagellin From Pseudomonas Aeruginosa Modulates SARS-CoV-2 Infectivity in CF Airway Epithelial Cells by Increasing TMPRSS2 Expression. bioRxiv (2020). 2020.08.24.264564. doi: 10.1101/2020.08.24.264564 PMC8688244 34950129

