# Supplementary material for: Flagellin From Pseudomonas aeruginosa Modulates SARS-CoV-2 Infectivity in Cystic Fibrosis Airway Epithelial Cells by Increasing TMPRSS2 Expression
Source: Front Immunol. 2021 Dec 7;12:714027. doi: 10.3389/fimmu.2021.714027 (PMC8688244; doi:10.3389/fimmu.2021.714027)
Supplement: Supplementary file 1 [file DataSheet_1.docx]

**Supplementary Figure 1. Effect of increasing concentration of *P. aeruginosa* flagellin on TMPRSS2 expression in Calu-3 cells.** *TMPRSS2* mRNA expression in Calu-3 cells (ATCC) grown at the air-liquid interface and stimulated for 6 h with *P. aeruginosa* flagellin (*Pa*-F, 0, 5, 50, 500 ng/mL) (*n* = 3, ANOVA with Bonferroni’s multiple-comparison test, ****P* < 0.001, *****P* < 0.0001). *GAPDH*, housekeeping gene*.*

**Supplementary Figure 2. Effect of *P. aeruginosa* flagellin on *ACE2* expression and cytokine production in Calu-3 cells.** Production of IL-8 (**A**) and IL-6 (**B**) by Calu-3-*CFTR*-WT and -*CFTR*-KD cells grown at the air-liquid interface and either not stimulated or stimulated for 3 or 6 h with *Pa*-F (50 ng/mL) (*n* = 5, ANOVA with Bonferroni’s multiple-comparison test, ***P* < 0.01, *****P* < 0.0001).

**Supplementary Figure 3. Effect of flagellins on *TMPRSS2* expression and IL-8 production in Caco-2/TC7 cells.** *TMPRSS2* mRNA expression (**A**) and IL-8 production (**B**) in Caco-2/TC7 cells stimulated for 6 h with 50 ng/mL *Pa*-F or *St*-F. ANOVA with Dunnett’s multiple-comparison test, *****P* < 0.0001.

**Supplementary Figure 4. Effect of flagellin on transepithelial resistance measurement (TEER) in *CFTR*-deficient Calu-3 cells.** TEER was measured before (0 h) and after stimulation for 16 h with 50 ng/mL *Pa*-F in Calu-3-*CFTR*-KD cells grown at the air-liquid interface. ANOVA with Bonferroni multiple-comparison test, **P* < 0.05, ****P* < 0.001.
